# The importance of perceived social support for symptoms of depression and academic stress among university students– A latent profile analysis

**DOI:** 10.1371/journal.pone.0324785

**Published:** 2025-05-23

**Authors:** Martyna Kotyśko, Joanna Frankowiak

**Affiliations:** Department of Clinical Psychology, Development and Education, Faculty of Social Sciences, University of Warmia and Mazury in Olsztyn, Olsztyn, Poland; Kazimierz Wielki University in Bydgoszcz: Uniwersytet Kazimierza Wielkiego w Bydgoszczy, POLAND

## Abstract

The aim of the study was to identify, among university students, profiles featuring severity of depressive symptoms, academic stress, and perceived social support and to compare the profiles by gender and current use of professional support. An online cross-sectional study of 1,519 college students in Poland was conducted between March and April 2021. Depressive symptoms were measured using the Center for Epidemiologic Studies Depression Scale—Revised (CESD-R), perceived social support was gauged with the Multidimensional Scale of Perceived Social Support (MSPSS), and academic stress was determined using a short scale of the author’s own design. The main analysis used in the study was latent profile analysis (LPA), which represents a person-centered approach. It allows researchers to identify the structure of the research sample due to the presence or severity of the variables included in the analysis. Four profiles emerged from the analysis: (1) low intensity of depressive symptoms and academic stress with social support (53%); (2) average intensity of depressive symptoms and academic stress with reduced social support from a significant other (19%); (3) high intensity of depressive symptoms and academic stress with low perceived social support (10%); and (4) high intensity of depressive symptoms and academic stress with support from a significant person (18%). Gender and current use of support were factors that significantly differentiated the profiles. Capturing the diversity of the subjects in terms of the severity of depressive symptoms and academic stress in the context of perceived social support provides an opportunity to gain a better understanding of their situation and, consequently, to take more appropriate measures to improve their situation.

## Introduction

Researchers are eager to explore the mental health of college students, assuming that they are a group particularly prone to experiencing problems, including increased symptoms of stress and depression [1]. The reasons are attributed to the peculiarities of the developmental period of early adulthood [2–5] and also to the challenges of the study process itself.

In recent years, a factor that significantly intensified academic stress and increased depressive symptoms among students was the outbreak of the COVID-19 pandemic [6–9]. According to a report on a survey conducted in 2020 in Poland by the Public Opinion Research Center (*Centrum Badania Opinii Społecznej,* CBOS), among respondents aged 18–24, “the frequency of experiencing depression, helplessness and weariness among the youngest respondents reached the highest level in the last two decades” [10]. Some researchers pointed to a nearly twofold increase [11]. As a natural consequence of such a state of affairs, it is natural to ask questions about protective factors against the negative impact of stress on mental health and the increase in depressive symptoms among students.

A factor that can significantly improve students’ well-being is the informal and formal support received from various sources [12]. Meta-analytic reviews have indicated that social support predicts better mental health functioning and may be considered a protective factor against the onset of mental health problems [13–15].

## Academic stress and symptoms of depression among students

Stress is inherent to student life due to the internal and external expectations and changes that occur at individual and societal levels following university admission. Academic stress is defined as the mental and emotional pressure or tension that occurs due to university requirements [16]. Research has confirmed that overexposure to academic stress can have negative consequences for mental health [17] and is significantly correlated with psychological well-being [18]. Researchers have also found a relationship between severe academic stress and depression [19–21].

Female students are more vulnerable to stress compared to men [8,18,19,22,23]. They also score higher on depressive symptoms [24–27]. In addition, severe stress in women significantly increases their risk of depressive symptoms [28].

Not insignificant for mental functioning was the COVID-19 pandemic and its third wave, which lasted from March to May 2021. That wave brought a significant increase in the number of cases and hospitalizations not only in Europe, but also in South America and Asia, due to the emergence of a new, more infectious variant of the virus, Delta, which also led to a more severe course of the disease. In Poland, a record was set on 1 April, with 35,251 new cases – the highest daily number since the beginning of the pandemic [29,30]. The highest bed occupancy rates were also reported [30]. Further restrictions were introduced to enforce social isolation, including the closure of hotels, cultural institutions, sports facilities, malls, and shopping centres to a limited extent. A decree issued on 26 February 2021 reinstated the obligation for universities to deliver classes remotely [31].

Research conducted at the time found that Polish students reported a decrease in life satisfaction and an increase in perceived anxiety as they aged [32]. Perceived stress, on the other hand, was higher during the first phase of the pandemic and decreased significantly in subsequent waves. Additionally, female gender was a significant risk factor for experiencing severe stress [32].

Researchers analysing changes in student functioning over the months of the pandemic observed a decrease in overall pandemic-related anxiety despite an increase in COVID-19-related illnesses and deaths. This decrease may have been a result of adaptation to the stressful situation, although the situation itself did not improve positively despite the passage of time [33,34]. However, the results of a study confirmed that young adults, compared to older adults, fared worse in facing the emotional and psychological effects of the COVID-19 pandemic in its third wave [35].

## College student support vs. stress and depressive symptoms

The literature uses the terms perceived and received support [36]. The former refers to the subjective assessment of how individuals perceive friends, family members, and others in the immediate environment as available to provide material, psychological, and general support. Received support, on the other hand, is measured by objective indicators and expresses the real support received. Researchers have indicated that perceived support is a more important determinant of health [37,38], compared to received support [39,40].

Emotional support, which provides a sense of safety and security in stressful situations, influences coping strategies and contributes to an individual’s well-being [41,42], is mainly significant, and promotes lower levels of stress [43] and depression [13,44,45]. Students with low perceived social support are five to six times more likely to experience depressive and anxiety symptoms than those with high perceived social support [25,46]. In general, students’ perceived social support has been associated with a reduced severity of depressive symptoms [25,47] and academic stress [20].

Of particular importance to students’ mental health is the emotional support of family [42], mitigating risks to their mental health [48]. Family support meets an individual’s needs for acceptance, belonging, and love [49] and is associated with students’ sense of their ability to cope with difficult experiences related to the study process [50]. In contrast, low parental support has been associated with a higher risk of academic stress [51] and has been significantly associated with levels of depression [47,52–54]. This is particularly applicable when perceived stress reactivity is low [55].

Peer support is also important for students’ psychological well-being [42]. Friends and acquaintances in early adulthood may be a more important source of support compared to family members [56], and their support is a significant predictor of depression [57]. Perceived peer support has negatively affected depressive symptoms, and both negative and positive indicators of emotional well-being mediated the relationship between perceived available peer support and depressive symptoms in Sun et al. [58]. Also, international studies conducted during COVID-19 confirmed the indirect effect of perceived social support from friends on the association between social loneliness and depression and indicated that support from friends significantly reduced depressive symptoms [47].

Given the high level of mental health problems among students, questions have been raised about the need to offer students psychological support as well as students’ willingness to take advantage of it. On the one hand, researchers have pointed to the importance of the social climate on campus and the need for institutional support; conversely, they have investigated students’ readiness to take advantage of institutional support, including professional psychological and psychiatric help [59]. Significantly higher readiness in this regard has been shown by women compared to men [60]. Also, a 2021 report on students’ mental health prepared by Niezależne Zrzeszenie Studentów (the Independent Students’ Association) and Polskie Stowarzyszenie Studentów i Absolwentów Psychologii (the Polish Association of Psychology Students and Graduates) [61], based on research with a sample of nearly 3,000 students from all over Poland, indicated that students rated the activities of universities as insufficient in terms of promoting and caring for the mental health of students.

In Poland, the issue of providing psychological support to students in the educational process has only recently been addressed. Psychological support can help students cope better with their studies and responsibilities. Psychological problems make it difficult to concentrate, stay motivated, and be systematic, and can therefore be conducive to dropping out of studies. Action on student mental health is also a kind of investment in society. We think of university graduates as people who represent the future of the country. Strengthening their health can and should also be motivated by paving the way for their well-being and self-fulfilment.

Different types of initiatives related to mental health promotion and emotional support have been aimed at students, including activities to level the playing field with regards to people with disabilities, and implemented in different academic environments in Poland [62]. Most often these are educational activities that spread knowledge about particular mental disorders and ways to support people who suffer from them, but also individual consultations and forms of group support have been provided [63]. An important impetus for discussing the need to expand provisions in this area was the COVID-19 pandemic, which brought a significant increase in mental health problems.

Based on the foregoing findings, it was reasonable to hypothesize that those students with high rates of perceived social support would report lower rates of depressive symptoms and experience less academic stress. In contrast, those in whom perceived social support was low were likely to experience greater academic stress and more severe depressive symptoms. We wondered whether these patterns would appear in the present study, and whether there might be other patterns between the variables. Overall, the main aim of this study was to identify groups among the students surveyed based on the severity of depressive symptoms, academic stress, and perceived social support, and to determine whether the profiles differed in terms of gender and current use of professional support. Specifically, the following research questions were formulated:

Q1: How many latent profiles can be identified in the sample of surveyed students?

Q2: What are the characteristics of the identified latent profiles in terms of the severity of the key variables in the study?

Q3: Do the profiles identified differ in terms of gender proportions?

Q4: Do the profiles identified differ in terms of the proportion of people currently receiving professional support?

## Materials and methods

### Procedure and participants

This study was conducted as part of the project “Research on the Demand for Psychological Support of University Students – 2021” (https://doi.org/10.17605/OSF.IO/P2Y84). Its implementation included four scientific objectives. One of these was to verify the structure of the Center for Epidemiologic Studies Depression Scale – Revised (CESD-R) in a sample of university students. This objective was met and the data published in Kotyśko and Frankowiak [64]. The last objective, related to the presented manuscript, assumed using a person-centred approach to identify clusters that included the following variables: academic stress, depressive symptoms, and perceived social support.

The facilitation of the project took place during the third wave of COVID-19 (recruitment period: 26 March 2021–28 April 2021), when remote-learning occurred at Polish universities. A total of 1,519 students from one Polish university took part in this study. The participants represented all faculties of the university (mean response rate among faculties: 9.86%, range: 2.86%–14.0%), assuming a survey of approximately 10% of the university student population.

The sample included students from different study years (full-time students only): 1st year of bachelor’s or long-cycle (*n* = 544; 38.8%); 2nd year of bachelor’s or long-cycle (*n* = 299; 19.7%); 3rd year of bachelor’s or long-cycle (*n* = 345; 22.7%); 4th year of long-cycle or 1st of second cycle (*n* = 200; 13.2%); 5th or 6th year of long-cycle or 2nd year of second cycle (*n* = 119; 7.8%); and lack of response (*n* = 12; 0.8%). There were more female (*n* = 1,172; 77.2%) than male (*n* = 347; 22.8%) participants. The current use of professional support (e.g., psychologist, therapist, psychiatrist) was reported by 133 participants (8.8%). The project received approval from the Scientific Research Ethics Committee of the University of Warmia and Mazury in Olsztyn.

The survey was conducted online in the Webankieta portal. Information about the survey, along with a link to the form, was disseminated via academic media and on the social networks of the various units of the university. Academics were also invited to collaborate and were asked to provide students with a link to the survey while students took online classes. The students at the beginning of the survey were informed about the research purpose, that participation in the study was voluntary and anonymous, they could opt out at any time, and the data would be used for research purposes only. Consent to participate in the study was given anonymously online by ticking a box entitled “I agree to participate in the study”.

### Measures

#### Depressive symptoms.

The Center for Epidemiologic Studies Depression Scale – Revised, CESD-R [65], Polish Adaptation by Koziara [66], was used to measure symptoms of depression in the general population and consists of 20 items to which the subject responds based on the following frequency scale: (0) *not at all or less than 1 day*; (1) *1–2 days*; (2) *3–4* days; (3) *5–7* days; and (4) *almost every day for 2 weeks.* Scores range 0–80. The higher the score, the higher the intensity of the depressive symptoms. In the analyses, a proposal for a shortened version of the tool prepared by Kotyśko and Frankowiak [64] was used, including nine items, with each item corresponding to one symptom of depression according to the DSM-IV-TR and DSM-5 classifications. This version obtained satisfactory parameters in the conducted confirmatory analyses and has been characterized by measurement invariance regarding gender [64]. Scores range 0–36. The Cronbach’s alpha for the version of the CESD-R-9 in this study was .87.

#### Perceived social support.

The Multidimensional Scale of Perceived Social Support (MSPSS) [67]; Polish Adaptation by Buszman and Przybyła-Basista [68], consists of 12 items which refer to three sources of support: family, friends, and a significant other. The participant responds to statements based on a 7-point Likert scale where 1 means *strongly disagree* and 7 means *strongly agree*. It is possible to calculate an overall score for all items (scores range 12–84), but the scale also takes into account three subscales according to the source of support – each with four items (for each, scores range 4–28). The higher the overall score and the subscale scores, the higher the perceived social support. The authors of the MSPSS scale in their study [67] reported the following Cronbach’s alpha coefficients: total score .88, Family .87, Friends .85 and Significant Other .91. In a Polish study by Buszman and Basista [68] coefficient values were as follows: total score .89, Family .92, Friends .93, and Significant Other .87. In the present study, the obtained alpha coefficients were very similar to those obtained in the mentioned Polish study: The overall score was .91, and for the individual subscales: Family (α = .92), Friends (α = .95), and Significant Other (α = .92).

#### Academic stress.

Due to pandemic circumstances, including those related to distance-learning, a short scale was created to assess academic stress. It included questions on aspects that changed due to the process of distance-learning and the lack of face-to-face contact (i.e., verifying knowledge), as well as contact with instructors and other university staff regarding the course of study. Nine items were developed and used in the administered survey. Survey participants were asked to indicate how stressful they found the given situations, using the following response scale: 0 meant *not stressful at all* and 5 meant *stressful to a very high degree*. Only the Academic Stress subscale related to the study process was used for the analysis (α = .84).

An exploratory factor analysis (EFA) was conducted, with consideration of equivalent factors. The principal axis method and oblimin rotation were used to reveal a latent item structure. The result is presented in Table 1 with factor loadings after rotation. Based on the KMO measure (.83), it could be concluded that the sample was adequately recruited. Bartlett’s test of sphericity was statistically significant (χ^2 ^= 5,154.97, df = 36, *p* < .001), confirming that there were significant relationships between the variables included. The two-factor solution allowed for 50.34% of the variance to be explained.

**Table 1:** Nine Items Included in the Measurement of Academic Stress and Exploratory Factor Analysis (method: principal axis; rotation: oblimin) with factorial loadings, *n* = 1,519.

Item phrasing	Factor 1	Factor 2
ENG: Taking written tests/exams PL: Zdawanie egzaminów/kolokwiów pisemnych	-.03	**.69**
ENG: Taking oral tests/exams PL: Zdawanie egzaminów/kolokwiów ustnych	-.05	**.65**
ENG: The scope of the required material PL: Zakres materiału, który trzeba opanować	.14	**.55**
ENG: Periods of high pressure (pre-session and session) PL: Okresy wysokiej presji (okres przed sesją i sesja)	.00	**.76**
ENG: Asking lecturers for help/advice PL: Proszenie wykładowców o pomoc/radę	**.83**	.03
ENG: Understanding lecturers’ expectations PL: Zrozumienie oczekiwań wykładowców	**.60**	.15
ENG: Contacting your year tutor PL: Kontakt z opiekunem roku	**.67**	-.06
ENG: Contacting a teacher who conducts one of your classes PL: Kontakt z osobą prowadzącą zajęcia	**.92**	-.05
ENG: Dealing with formal matters at the dean’s office/faculty regarding your studies PL: Załatwianie spraw formalnych w dziekanacie/na wydziale dotyczących twoich studiów	**.57**	-.01

*Note*. Values in bold indicate the assigned factor.

On the basis of the analysis, it was assumed that the first factor determined academic stress related to examinations and included four items (α = .75), while the second factor concerned academic stress related to the study process, and it was made up of five items (α = .84). The higher the score on a scale, the higher the severity of academic stress in the area studied. Accounting for the practical aspect of the research being carried out, we decided to include as an indicator in the latent profile analysis only the subscale relating to the study process, which universities can influence during distance-learning.

#### Sociodemographic data and current use of professional support.

A brief self-report survey was used to collect information about participants’ gender (*women *= 1, *men *= 2), faculty (chosen from a provided list of 16 faculties), and year of study (chosen from a provided list: 1st year of bachelor’s or long-cycle, 2nd year of bachelor’s or long-cycle, 3rd year of bachelor’s or long-cycle, 4th year of long-cycle or 1st of second cycle, 5th or 6th year of long-cycle or 2nd year of second cycle). The participants were asked if they were currently receiving professional support provided by, for instance, a psychologist, therapist, or psychiatrist (coded as *yes* = 1, *no* = 0).

### Data analysis

An EFA with the principal axis method and oblimin rotation was used for initial analysis of the Academic Stress scale. Cronbach’s alpha coefficients and descriptive statistics were obtained. The values of skewness and kurtosis formed the basis for deciding whether the distribution of the study variables was normal. We assumed that the values of both indices should be within +/- 1.5 to conclude that there was no deviation from normality [69]. Pearson correlation coefficient was used in the analysis of relationships between variables. For the mentioned analyses, IBM’s SPSS v.29 was used.

Latent profile analysis (LPA) was the main analysis carried out and was performed in the R environment (Version 4.4.2; [70]) using the *tidyLPA* [71] and *dplyr* [72] packages. Solutions were tested for four models implemented in the *tidyLPA* package:

a) Model 1, assuming equal variances between profiles and zero covariances,
b) Model 2, assuming varying variances and zero covariances,
c) Model 3, assuming equal variances and equal covariances, and
d) Model 6, assuming varying variances and varying covariances [71].

The analysis was performed on standardized data. The following fit indices were used as the basis for selecting a solution with a specific number of classes/profiles:

(a) Akaike information criterion (AIC) and Bayesian information criterion (BIC), where the lower values indicated better fits of the proposed solutions [73].
(b) Bootstrap likelihood ratio test (BLRT) *p* value – “indicates if the *k*–1 class model is rejected in favor of the *k* class model” [73, p. 538].
(c) Entropy – the entropy value considered desirable should be greater than .8 [74].
(d) Minimum probability of assignment to a given profile (*prob_min*) which is “Minimum of the diagonal of the average latent class probabilities for most likely class membership, by assigned class. The minimum should be as high as possible, reflecting greater classification certainty (cases are assigned to classes they have a high probability of belonging to; see Jung & Wickrama, 2008)” [75, Section: Getting fit statistics].
(e) Minimum number of observations per class (N_Min) in a given solution [75].

Emerged profiles were compared in IBM’s SPSS v.29 with a Kruskal-Wallis ANOVA with post hoc tests (i.e., Dunn’s test with Bonferroni correction). Effect sizes for these nonparametric analyses were calculated with the usage of formulas implemented by Lenhard and Lenhard [76] in their effect size calculator, which transforms *H* statistics into eta square values (η^2^). Effect sizes for eta squares were interpreted according to Cohen [77] .01 = small; .06 = medium, and .14 = large effect. The relationship of profile assignment to gender and current use of support was tested with the chi-square test of independence (i.e., differences in proportions were analysed with a *Z*-test with Bonferroni correction). Cramer’s *V* was used as an effect size for the chi-square test of independence and interpreted according to Cohen [77], where effect size values are interpreted as .1 = small, .3 = medium, and .5 = large.

## Results

Based on the skewness and kurtosis values, we assumed that all variables were normally distributed. The Pearson correlation analysis conducted (Table 2) revealed significant associations between depressive symptoms, academic stress, and all types of perceived social support. Regarding the relationship between depressive symptoms and academic stress, the correlation coefficient was positive, while the aforementioned two variables correlated negatively with perceived social support.

**Table 2:** Descriptive statistics and correlations among the variables analysed.

	Variable	*M*	*SD*	Range	Skew.	Kurt.	1	2	3	4
1	DS	16.11	8.96	0–36	.16	-.86	–			
2	AS	9.60	5.93	0–25	.35	-.62	.37^**^	–		
3	PSS-FAM	18.72	6.65	4–28	-.51	-.66	-.39^**^	-.20^**^	–	
4	PSS-FR	20.55	6.39	4–28	-.90	.08	-.22^**^	-.19^**^	.36^**^	–
5	PSS-SO	22.07	6.41	4–28	-1.17	.52	-.17^**^	-.15^**^	.37^**^	.54^**^

*Note.* Skew. = skewness; Kurt. = kurtosis; DS = depressive symptoms; AS = academic stress; PSS-FAM = perceived social support, family; PSS-FR = perceived social support, friends; PSS-SO = perceived social support, significant other.

*p < .05.

**p < .01.

Next, an LPA was performed in which models considering one to six profiles were compared with each other (Table 3). Only for Model 1, assuming equal variances between profiles and zero covariances, the full analysis could have been prepared. Regarding Model 2, only solutions up to two profiles could have been provided. In Model 3, the analysis proceeded with the presence of system warnings and low entropy and very low minimum mean probability. For Model 6, similar to Model 2, only solutions with one or two profiles were provided. According to Akogul and Erisoglu [78] the best fit was noted for Model 6, with two profiles. Given the limited results obtained for models 2, 3, and 6, we decided to seek a solution in the region of Model 1.

**Table 3:** Model Fit Indices for Solutions Including One to Six Profiles Within the LPA for Models 1, 2, 3, and 6.

Model	Classes	AIC	BIC	Entropy	Prob_min	Prob_max	N_min	N_max	BLRT_*p*
1	One	21568.67	21621.93	1.00	1.00	1.00	1.00	1.00	–
	Two	20448.26	20533.47	.89	.90	.99	.18	.82	.01
	Three	19991.60	20108.77	.75	.80	.93	.15	.56	.01
	Four^*^	19735.54	19884.66	.80	.77	.94	.10	.53	.01
	Five	19701.69	19882.77	.71	.52	.91	.09	.42	.01
	Six	19538.26	19751.29	.74	.50	.92	.08	.44	.01
2	One	21568.67	21621.93	1.00	1.00	1.00	1.00	1.00	–
	Two	19765.84	19877.68	.78	.94	.94	.43	.57	.01
3	One	20268.85	20375.36	1.00	1.00	1.00	1.00	1.00	–
	Two^W^	20282.09	20420.56	.37	.00	1.00	.00	1.00	1.00
	Three^W^	19932.29	20102.72	.54	.00	.97	.00	.80	.01
	Four^W^	19895.71	20098.09	.51	.00	.90	.00	.53	.01
	Five	19728.82	19963.15	.46	.28	.94	.16	.24	.01
	Six	19814.10	20080.39	.48	.05	.92	.03	.38	1.00
6	One	20268.85	20375.36	1.00	1.00	1.00	1.00	1.00	–
	Two	19264.02	19482.38	.74	.91	.93	.37	.63	.01

Note: Variables in the model: depressive symptoms, academic stress, perceived social support from family, friends, and a significant other. AIC = Akaike information criterion; BIC = Bayesian information criterion; N_Min = minimum number of observations per class in a given solution; BLRT_*p* = bootstrap likelihood ratio test, *p*-value.

*Model selected as best;

^W^Solution with warning.

For Model 1, the AIC and BIC values were lowest for the solution with six profiles, while the entropy was highest for the solution with two profiles. The highest minimum probability was noted for the solutions with two and three profiles. Although the AIC and BIC parameters and the BLRT *p*-value indicated the choice of a solution with six profiles, the decision was made to choose a solution with four profiles. This solution, compared to those with a greater number of extracted profiles, had a higher entropy and minimum probability, which are related to certainty of classification. In addition, the four-profile solution fit into a model of prevention considered at three levels: universal, selective, and indicative (with the inclusion of two profiles).

**Fig 1:**
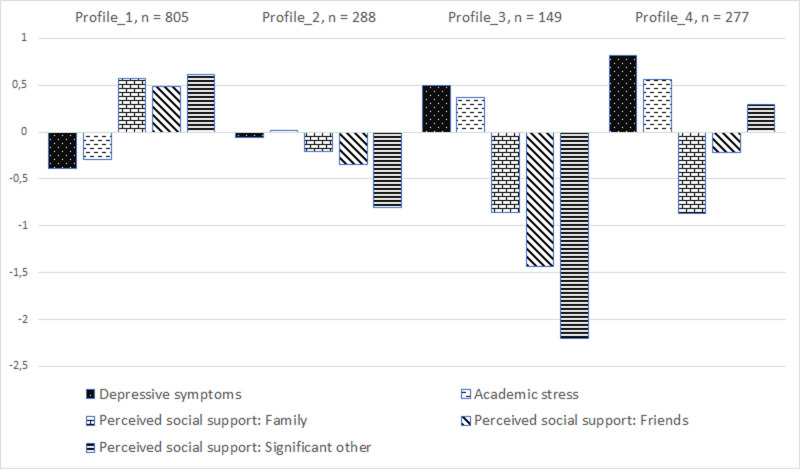
Graph of standardized averages for the four profiles selected in the LPA.

The following emerged profiles were characterized according to the intensity of the variables and levels of prevention (Fig 1):

- Profile 1 = low intensity of depressive symptoms and academic stress, with social support. This profile fit the largest group of students (53%, *n* = 805). Individuals in this profile can be considered recipients of activities at the level of so-called universal prevention oriented toward strengthening well-being and resources.
- Profile 2 = average intensity of depressive symptoms and academic stress, with reduced social support from a significant other. Individuals making up this profile represented 19% of the total sample (*n* = 288). People in this profile can be defined as recipients of selective prevention activities. These were people who developed depressive symptoms, and it would be worthwhile to target them with actions aimed at preventing the problem from developing (i.e., reducing the intensity of risk factors and strengthening protective factors).
- Profile 3 = high intensity of depressive symptoms and academic stress, with low perceived social support. This profile was the least common, including less than 10% of the students surveyed (*n* = 149).
- Profile 4 = high intensity of depressive symptoms and academic stress, with support from a significant person (*n* = 277). This profile, although largely similar to Profile 3, was characterized by having an above-average intensity of support from a significant other out of the three sources of perceived support with the other two sources at or below average levels.

In both profiles (3 and 4) were the recipients of so-called indicated prevention activities. In the case of elevated symptoms of depression and academic stress, priority should be given to measures that prevent these problems from worsening.

The profiles that emerged were compared with each other (using a Kruskal-Wallis ANOVA) in terms of the presence of differences within the variables used in the study, that is, depressive symptoms, academic stress, and the three types of perceived social support (Table 4). The results of the main test were statistically significant in each case, confirming the presence of differences between the profiles (the η^2^ effect sizes ranged from .13 to .67, which can be interpreted as medium and large effect sizes). A post hoc analysis showed that all four profiles differed significantly from each other per the level of depressive symptoms and the perceived social support of the significant other. No significant differences were noted between profiles 3 and 4 in terms of academic stress and perceived family support. Profiles 2 and 4 did not differ in terms of the level of perceived support from friends.

**Table 4:** Comparison of profiles in terms of the level of variables studied.

	Profile 1		Profile 2		Profile 3		Profile 4				
	Mean (SD)	Median (Range)	Mean (SD)	Median (Range)	Mean (SD)	Median (Range)	Mean (SD)	Median (Range)	H	η^2^	Post hoc
Depressive symptoms	12.54 (7.69)	12 (0-36)	15.39 (8.13)	16 (0-36)	20.68 (8.7)	21 (0-36)	24.74 (6.08)	25 (9-36)	422.21***	0.28	All sig.
Academic stress	7.80 (5.22)	7 (0-24)	9.79 (5.71)	10 (0-25)	11.77 (5.95)	12 (0-25)	13.44 (5.88)	14 (1-25)	196.18***	0.13	1-2***, 1–3***, 1–4***, 2–3*, 2–4***, 3–4ns
Perceived social support: Family	22.51 (4.52)	23 (4-28)	17.18 (5.45)	18 (4-27)	13.09 (6.34)	12 (4-26)	12.32 (5.36)	12 (4-28)	628.83***	0.41	1-2***, 1–3***, 1–4***, 2–3***, 2–4***, 3–4ns
Perceived social support: Friends	23.66 (4.35)	24 (4-28)	18.29 (4.88)	19 (4-28)	11.17 (6.07)	10 (4-28)	18.91 (6.41)	20 (4-28)	518.91***	0.34	1-2***, 1–3***, 1–4***, 2–3*, 2–4ns, 3–4***
Perceived social support: Significant other	26.02 (2.3)	27 (20-28)	16.66 (2.7)	17 (8-21)	7.69 (2.68)	8 (4-14)	23.93 (2.77)	24 (18-28)	1023.68***	0.67	All sig.

Note: H – Kruskal-Wallis test statistic; η^2^ - eta squared effect size value; Post hoc – Dunn’s test with Bonferroni correction; All sig – all comparisons significant at *p *< .05;

*p < .05; **p < .01; ***p < .001

In profiles 1 and 4, the percentage of women was significantly higher than in profiles 2 and 3. In Profile 1, the percentage of people currently using support was more than twice as low (6.7%) than in Profile 4, where the number of people who declared the use of support was almost 14.5% (Table 5).

**Table 5:** Comparison of profiles by gender and current use of professional support.

Variables	Profile 1, *n* (%)	Profile 2, *n* (%)	Profile 3, *n* (%)	Profile 4, *n* (%)	*Χ* ^2^	df	*p*	*V*
Gender								
Women	650^a^ (80.7)	191^b^ (66.3)	92^b^ (61.7)	239^a^ (86.3)	58.24^***^	3	<.001	.2
Men	155^a^ (19.3)	97^b^ (33.7)	57^b^ (38.3)	38^a^ (13.7)				
Current use of professional support								
Yes	54^a^ (6.7)	24^a,b^ (8.3)	15^a,b^ (10.1)	40^b^ (14.4)	15.81^**^	3	.001	.1
No	751^a^ (93.3)	264^a,b^ (91.7)	134^a,b^ (89.9)	237^b^ (85.6)				

*Note.* Values in rows marked with different letters are significantly different from each other at the *p* < 0.05 level.

*p < .05.

**p < .01.

***p < .001

## Discussion

Against the backdrop of research conducted during COVID-19, which mainly served to identify the percentage of students experiencing symptoms of depression and stress, our study used a person-oriented approach. The use of LPA (latent profile analysis) in our research made it possible to group subjects by similarity in terms of the intensity of variables studied while simultaneously keeping the differences between the emerged profiles separate. A nuanced picture of the study sample then emerged, as opposed to using a purely model-based approach where the relationships between variables play the most significant role. LPA had been successfully used in other studies performed with university students in Poland in relation to issues of behavioural addictions [79], academic burnout [80] or mental health [81].

Thus, a novelty of the present study was our inclusion of three types of perceived social support considered a protective factor in the LPA, in addition to depressive symptoms and academic stress.

The general results confirmed the existence of a positive relationship between experienced academic stress and depressive symptoms [20,21] and a negative relationship between perceived social support and depressive symptoms [25,47] and academic stress [20].

According to the first research question related to the number of profiles which could be identified, our LPA revealed that four original profiles could be extracted. Regarding the characteristics of the individual profiles according to key variables used in the study, in two profiles the pattern of key variables was as predicted; these included Profile 1 (low intensity of depressive symptoms and academic stress, with social support) and Profile 3 (high intensity of depressive symptoms and academic stress, with low perceived social support), where we observed the trend that experiencing perceived social support was associated with a reduced severity of both depressive symptoms [25,47] and academic stress [20]. Difficulties in coping with stress occurred together with experiencing apathy and depression also in a study by Frankowiak and Kotyśko [81], who also used LPA. Profile 1 in this text can be referred to as the Not Needing Support profile, while Profile 3 can be referred to the three profiles In Crisis, Increased Risk (i.e., the presence of suicidal thoughts or behaviors [IR-S]), and Increased Risk Without Suicidal Thoughts or Behaviors (IR-NS) per Frankowiak and Kotyśko [81].

Conversely, Profile 2 (average intensity of depressive symptoms and academic stress, with reduced social support from a significant other) and Profile 4 (high intensity of depressive symptoms and academic stress, with support from a significant person) showed an unexpected arrangement of variables. In Profile 2, when perceived social support was close to the mean score or slightly lower, levels of depressive symptoms and academic stress also oscillated around the mean. Despite the lower perceived social support from a significant other, people fitting this profile were not characterized by higher rates of academic stress and depressive symptoms. Parental support may have been significant in this case, which was at a level close to average in this profile. This may confirm the regularity captured in some studies that parental support is crucial for students’ well-being [42,47].

Profiles 3 and 4 needed to be studied more closely because of the elevated depressive symptoms. They had low perceived family support in common. This reflected a trend that low social support, including parental support, is associated with elevated rates of depressive symptoms [47,54] and academic stress [51]. Profiles 3 and 4, conversely, differed in terms of the level of perceived social support from friends and a significant person. It was surprising that Profile 4, despite reporting higher levels of support from friends and a significant other compared to Profile 3, reported the highest levels of depressive symptoms and academic stress. The above-average level of perceived social support from a significant other in this profile may indicate that those experiencing elevated symptoms of academic stress and depressive symptoms express a greater willingness to seek support, including from outside their circle of closest people.

The third research question regarded gender proportions in extracted profiles. The highest proportion of women was in profiles 1 and 4. The data in Profile 4 are consistent with researchers who found that women are significantly more likely to experience symptoms of academic stress [18] and depression [24,25].

The last research question concerned whether the profiles differed in terms of the proportion of people receiving support. The percentage of those receiving professional support in Profile 4 was significantly higher than among those who received support from family, friends, and a significant other (Profile 1). Although the two profiles were also characterised by a similar gender ratio, the higher number of people receiving support present in Profile 4 may have its origins in the tendency for women to seek support more often compared to men [60]. From this perspective, the meaning of the support from a special person in relation to the experienced depressive symptoms and academic stress is a topic that requires further exploration. The distinct pattern of variables in Profile 4 begs for hypotheses about who the most significant supportive others are and whether these supports include professionals, like therapists or psychologists. Furthermore, do these individuals appear in the lives of study participants as a result of the participants’ increased depressive symptoms and academic stress?

The analysis carried out allowed four profiles to emerge, grouping people according to the severity of their stress and depressive symptoms and perceived social support. The identified groups can be treated as recipients of preventive measures according to the severity of the problems experienced and the actions that should be taken with them. Such an approach can be helpful in designing countermeasures. People in Profile 1 were the recipients of activities at the level of so-called universal prevention oriented towards enhancing well-being and resources. The recipients of selective prevention activities were those assigned to Profile 2. These were people with depressive symptoms, and it would be worthwhile to target them with activities aimed at preventing the problem from developing (i.e., reducing risk factors and strengthening protective factors). In addition to actions oriented towards acquiring competences for coping with academic stress, all kinds of actions oriented towards strengthening relationships within academic communities and integration activities can be helpful for this group.

Particular attention should be paid to people in profiles 3 and 4. For them, measures from the level of indicative prevention should be addressed, aimed at preventing the worsening of symptoms of stress and depression. Access to both psychological and psychiatric support is becoming increasingly important. Both profiles showed insufficient perceived parental support, which is important for mental health. Its absence can be linked to various other problems in these relationships. In addition, in Profile 3 were those to whom help in the form of support groups could be addressed. This is a formula that promotes better coping with problems, also by establishing relationships with other people in a similar situation and at the same time providing a source of mutual emotional support.

The results of the present research confirm the need for various measures. They should all be oriented primarily towards enhancing protective factors. In the context of the presented research results, it seems reasonable to strengthen environments conducive to establishing relationships both at the campus level and at the level of individual academic programs or student groups. Universities should work to reduce academic stress.

Also of importance are initiatives oriented towards strengthening protective factors (i.e., knowledge and skills important for preventing the emergence and worsening of mental health problems, including depression). High rates of depressive symptoms and inadequate perceived parental support should lead to widening the availability of psychological support for students, paving the way for the possibility of psychiatric consultation.

## Limitations

The survey we conducted was not free from limitations. Participants completed questionnaires that were self-report in nature (i.e., reflecting only subjective perceptions of themselves and their own situation). The period of the study, which fell during the third wave of the COVID-19 pandemic, is also significant. It seems necessary to repeat the study in a different period and re-verify the number and specificity of the emerged profiles. Also, the sample was not representative and balanced in terms of gender and year of study. As the study was conducted at a single university, its specificity may also have influenced the results obtained and thus limits the results’ generalizability.

## Conclusion

This study was conducted with LPA and was able to provide a holistic picture of the situation of the students surveyed with the variables of depressive symptoms and academic stress and the three types of perceived social support. The diversity of the students was shown, which is difficult to capture if only linear relationships between variables are considered. As a results four profiles were identified with an LPA. Profiles 1 and 3 had a pattern consistent with the predictions: Experiencing social support was associated with a reduced severity of depressive symptoms and academic stress. The other two profiles completed the picture of variation in student functioning due to the presence or absence of a specific form of support in relation to experienced academic stress and depressive symptoms.

The use of LPA allowed for a more informed design of broad prevention activities tailored to the needs of individual recipients. This is another argument in favour of the need for comprehensive measures aimed at students. So far, Poland lacks systemic solutions at universities, which translates into piecemeal actions dedicated to a narrow group of students identified as most in need of support in connection with problems which hamper the process of studying.
